# Miscellaney

**Published:** 1896-10

**Authors:** 


### Miscellany.

Climate and Health, a publication of the weather bureau of the United States department of agriculture, has been discontinued, owing to the failure of Congress to make the necessary appropriation.

---

Paris has 521 foreign medical men among the 2,922 in the city. It is supposed that New York has more than 600 native American medical men among its 3,000 practitioners.—*Medical Herald.*

---

The Legal Status of the Skiagraph.—A Boston court has ruled out X-ray photographs of injuries to a boy's head, which figured as exhibits in a $50,000 suit for damages. Corporations will probably file away reports of this decision in the case of Renford vs. Rogers for future use.—*Journal of Nervous and Mental Diseases.*

---

Brains.—This organ of man is now in special request in Boston, the hub-city of brains. They are wanted for scientific study—for what special purpose or what end in view is not stated. Before long we may see a like demand for gastrocnemii, to determine how long one had used the wheel. No limit of field to scientific inquiry!—*Medical Herald.*

